# Rotational Head Kinematics in Football Impacts: An Injury Risk Function for Concussion

**DOI:** 10.1007/s10439-011-0392-4

**Published:** 2011-10-20

**Authors:** Steven Rowson, Stefan M. Duma, Jonathan G. Beckwith, Jeffrey J. Chu, Richard M. Greenwald, Joseph J. Crisco, P. Gunnar Brolinson, Ann-Christine Duhaime, Thomas W. McAllister, Arthur C. Maerlender

**Affiliations:** 1 School of Biomedical Engineering & Sciences, Virginia Tech-Wake Forest University, 440 ICTAS Building, Stanger St, Blacksburg, VA 24061 USA; 2 Simbex, Lebanon, NH USA; 3 Thayer School of Engineering, Dartmouth College, Hanover, NH USA; 4 Department of Orthopaedics, The Warren Alpert Medical School of Brown University and Rhode Island Hospital, Providence, RI USA; 5 Edward Via Virginia College of Osteopathic Medicine, Blacksburg, VA USA; 6 Pediatric Neurosurgery, Dartmouth Hitchcock Medical Center, Hanover, NH USA; 7 Department of Psychiatry and Neurology, Dartmouth Hitchcock Medical School, Lebanon, NH USA; 8 Department of Psychiatry, Dartmouth Hitchcock Medical Center, Hanover, NH USA

**Keywords:** Mild traumatic brain injury, Head, Helmet, Angular, Acceleration, Sports, HITS

## Abstract

Recent research has suggested a possible link between sports-related concussions and neurodegenerative processes, highlighting the importance of developing methods to accurately quantify head impact tolerance. The use of kinematic parameters of the head to predict brain injury has been suggested because they are indicative of the inertial response of the brain. The objective of this study is to characterize the rotational kinematics of the head associated with concussive impacts using a large head acceleration dataset collected from human subjects. The helmets of 335 football players were instrumented with accelerometer arrays that measured head acceleration following head impacts sustained during play, resulting in data for 300,977 sub-concussive and 57 concussive head impacts. The average sub-concussive impact had a rotational acceleration of 1230 rad/s^2^ and a rotational velocity of 5.5 rad/s, while the average concussive impact had a rotational acceleration of 5022 rad/s^2^ and a rotational velocity of 22.3 rad/s. An injury risk curve was developed and a nominal injury value of 6383 rad/s^2^ associated with 28.3 rad/s represents 50% risk of concussion. These data provide an increased understanding of the biomechanics associated with concussion and they provide critical insight into injury mechanisms, human tolerance to mechanical stimuli, and injury prevention techniques.

## Introduction

There are an estimated 1.6 to 3.8 million sports-related concussions occurring annually in the United States.^25^ While sports-related concussion was once considered to only result in transient symptoms and neurocognitive impairment, recent research has raised the possibility of links between repetitive concussions and neurodegenerative processes in some athletes.^12^,^37^,^38^ Such reports have increased awareness and media attention on the potential health risks of concussions. This paper focuses on the biomechanics of the head associated with sports-related concussion. An increased understanding of these concussive biomechanics may provide insight to the injury mechanisms, human tolerance to mechanical stimuli, and injury prevention techniques.

Traumatic brain injury (TBI) occurs across a spectrum of severity with sports-related concussion falling at the mild end of this spectrum. Historically, the majority of brain injury biomechanics research has focused on moderate and severe TBI of various types, including focal and diffuse injuries. Concussive brain injury is unique in that the injury has a graded response that can vary from minor confusion to death. However, the varying grades of concussion are likely a scaled result of the varying mechanical stimuli input to the head.^39^ Previous work has explored how kinematics of the head, presumably indicative of the inertial response of the brain, relate to diffuse brain injury mechanisms. Ideally, the head kinematics of a human surrogate could be measured in a safety testing scenario and used to predict the tissue level response of the brain in an effort to evaluate injury potential. With this goal in mind, many researchers have studied the relationship between head kinematics and brain injury. Most experiments have investigated linear or rotational kinematics independently, as these inputs have long been thought to result in different pathoanatomic injury types.^52^ Explanations of these theories have been previously documented in great detail.^20^

The Wayne State Tolerance Curve (WSTC) was developed from a series of tests on dogs and cadavers and related linear acceleration and duration of acceleration to injury tolerance.^16^ Injury metric functions such as severity index (SI) and head injury criterion (HIC) were subsequently developed from analyses of the WSTC.^11^,^53^ These injury metrics were primarily developed to predict skull fracture, although they were thought to likely correlate with severe parenchymal brain injury as well. Notably, only linear acceleration is considered in these injury metrics, and all current safety standards for head injury are based on these works. However, rotational acceleration is believed by many to be a primary mechanism for diffuse brain injury, including loss of consciousness and concussion.^23^ Unlike linear acceleration, there is currently no accepted injury criterion for rotational acceleration. Additionally, previous research investigating rotational kinematics has focused on animal models (primate or rat), in which pure rotational acceleration was applied to the head.^8^,^13^,^14^,^27^,^28^,^39^,^40^ These experiments, including those evaluating linear and rotational acceleration, utilize little data from humans. Cadavers have no physiologic response, and animal data cannot be directly applied to humans. Optimally, these experiments would utilize data derived from humans. However, recording potentially injurious data from humans has been challenging. One relatively recent approach has been to use contact sport athletes, a group at elevated risk for sustaining concussions, to characterize the biomechanics of this specific injury type.

Of all sports, football has the greatest incidence of concussion due to its large number of participants and its high rate of head impact events.^22^ The high incidence of concussion in football provides a unique opportunity to collect injury related biomechanical data. With this in mind, a series of studies reconstructed concussive impacts experienced by players in the National Football League (NFL) was performed using Hybrid III anthropometric test devices (ATD).^34^,^35^,^42^ Using game film, 31 impacts were reconstructed and the resulting head kinematics were analyzed. From these analyses, separate injury risk curves for concussion were developed for linear and rotational kinematics. The limitations of this study were that data were collected from ATDs rather than humans, and that the NFL dataset did not quantify head impact exposure.

More recently, researchers have instrumented and observed a population that is at high risk for concussion (football players) to collect head impact data at potentially injurious severities from human volunteers in a natural and ethically sound manner.^10^ In these studies, the helmets of football players were instrumented with commercially available accelerometer arrays, known as the Head Impact Telemetry (HIT) System (Simbex, Lebanon, NH). Each time an instrumented player’s helmet was impacted, head acceleration data were recorded and stored. This method of data collection allows biomechanical data measured in humans to be paired with clinical data assessing injury. These studies have provided insight into the head kinematics associated with head impacts in football, but have largely been descriptive studies with small concussive sample sizes making it difficult to draw conclusions about injury.^3^,^4^,^9^,^17^,^33^,^44^,^48^

Using a large head acceleration dataset collected from human volunteers, the objective of this study was to characterize tolerance to the rotational kinematics resulting from helmeted head impacts associated with sports-related concussion. Impact distribution models and descriptive statistics for sub-concussive and concussive impacts are provided. Furthermore, a new injury risk function has been developed through a logistic regression analysis that considers injury incidence rates. Data presented in this study provide valuable insight to the concussive tolerance of humans to rotational acceleration.

## Materials and Methods

### Data Collection

Between 2007 and 2009, the helmets of 335 collegiate football players were instrumented with accelerometer arrays that measured head acceleration for every head impact each player experienced. Players were recruited from three Division 1 National College Athletic Association (NCAA) football teams (Brown, Dartmouth, and Virginia Tech), and all participants gave informed consent approved by each school’s Institutional Review Board (IRB). Two accelerometer arrays were utilized in this study: the commercially available HIT System and a custom 6 degree of freedom (6DOF) measurement device.

**Figure 1:**
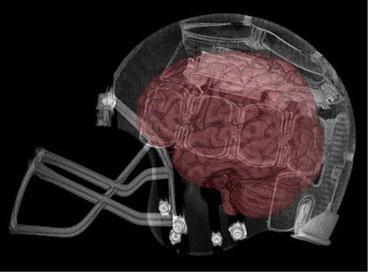
CT scan of an instrumented helmet merged with an MRI of a human brain demonstrating how the accelerometer array fits between the normal padding of football helmets

A total of 314 players were instrumented with the HIT System for every game and practice they participated in while included in this study (Fig. 1). The HIT System consists of six accelerometers that are mounted on a specifically designed elastic base so that they remain in contact with the head at all times, ensuring that head acceleration is measured rather than shell vibrations.^26^ When an accelerometer exceeded a specified threshold (14.4 g) during play, data acquisition was automatically triggered and data were collected for 40 ms (including 8 ms of pre-trigger data) at 1000 Hz. Once data collection was complete, data were wirelessly transmitted to a computer on the sideline. Resultant linear head acceleration at the center of gravity (CG) of the head was computed using a novel algorithm.^6^ The HIT System has been well-validated,^6^,^9^ and has been widely adopted by other researchers studying concussion in athletes.^10^ This study utilized data collection protocols that are described in greater detail by previous studies.^7^,^9^

In addition, the helmets of 21 Virginia Tech football players were instrumented with a custom 6DOF head acceleration measurement device.^44^ This measurement device was similar to the standard HIT System, but consisted of 12 accelerometers that were positioned and oriented in a different manner. Linear and rotational acceleration about each axis of the head is computed using a novel algorithm.^5^,^43^ While an overview is presented here, a detailed technical comparison of the HIT System and 6DOF measurement device has previously been reported.^43^

Measured impacts were categorized as either being sub-concussive or concussive. For the purposes of this study, concussion was defined as an alteration in mental status resulting from a blow to the head, which may or may not involve loss of consciousness. Using the guidelines set forth by the summary and agreement statement of the Second International Conference on Concussion in Sport in Prague,^31^ concussions were diagnosed by each team’s trained medical staff from signs, symptoms, computer-based neurocognitive testing, and clinical judgment. Symptoms associated with concussion included: headache, nausea, vomiting, dizziness/balance problems, fatigue, trouble sleeping, drowsiness, sensitivity to light or noise, blurred vision, difficulty remembering, and/or difficulty concentrating.^31^ The time of concussion diagnosis varied from immediately after the impact associated with injury -to- later that day -to- days after the injury when the athlete self-reported symptoms or signs of concussion were observed by the medical staff. Following diagnosis of concussion, anecdotal observations about the injury (suspected time of injury, a description of the impact, and other comments) from the player, coaches, and trainers was combined with video of the event and biomechanical data to associate the injury with a single head impact. All other head impacts recorded were labeled sub-concussive. To increase the sample size of the concussive dataset, the concussive impacts measured in this study were compiled with concussive data collected from published studies that utilized similarly reported data collection methods and guidelines for the diagnosis of concussion.^3^,^17^

### Data Analyses

Linear acceleration was directly measured by the HIT System as previously described. Traditionally with the HIT System, peak rotational acceleration has been estimated from the linear acceleration vector and an assumed point of rotation 10 cm inferior to the head CG. In this study, peak rotational acceleration was estimated using Eq. (1), which was derived from the equations of motion modeling a force acting on the head; where α is peak rotational acceleration, *m* is the mass of the head, *ax* is peak linear acceleration along the anterior–posterior axis of the head, *ay* is peak linear acceleration along the medial–lateral axis of the head, *I* is the moment of inertia of the head, and *d* is the perpendicular distance from the head CG to the impact vector. The unknown parameters (*m*, *I*, and *d*) of Eq. (1) were combined into a single variable, which was determined through a regression model analysis of recorded 6DOF acceleration data, and confirmed with laboratory validation experiments similar to those previously reported.^43^ A least squares technique was used to solve for the combined variable (*m* × *d*/*I*), which was determined to be 6.48 m^−1^. Peak rotational accelerations were determined for all recorded HIT System impacts using Eq. (1).

1 $$ \alpha = \frac{{m\sqrt {ax^{2} + ay^{2} } }}{I}d $$

Each recorded head impact was categorized into one of four general impact locations: front, rear, side (left and right), and top.^7^ Impacts to the left and right locations were assumed symmetric, and thought to invoke coronal plane rotation. Impacts to the front and back of the helmet were grouped to together and thought to invoke sagittal plane rotation. Impacts to the top of the helmet have been shown to primarily cause linear events, as the head loaded is in line with the cervical spine. For this reason, impacts to the top of the helmet were removed from this analysis and reported separately.

The data collected in this study were used to define the overall distribution of sub-concussive and concussive impacts with relation to rotational acceleration. Sub-concussive impacts recorded using the HIT System and 6DOF measurement device were each fit to Weibull distributions. These data were fit to Weibull distributions because the acceleration distributions of sub-concussive impacts were highly right-skewed. The Weibull probability density function (pdf) takes the form of Eq. (2), while the Weibull cumulative density function (cdf) takes the form of Eq. (3). For these equations, *x* is the peak resultant rotational acceleration, α is the shape parameter, and β is the scale parameter. Weibull distribution parameters were estimated using a maximum likelihood technique.

2 $$ w_{\text{pdf}} = \frac{{\alpha \left( x \right)^{\alpha - 1} }}{{\beta^{\alpha } }}e^{{ - \left( {\frac{x}{\beta }} \right)^{\alpha } }} $$

3 $$ w_{\text{cdf}} = 1 - e^{{ - \left( {\frac{x}{\beta }} \right)^{\alpha } }} $$

Concussive impacts collected with the HIT System were fit to a Rician distribution, which is a form of a normal distribution that is non-negative. The Rician pdf takes the form of Eq. (4), while the Rician cdf takes the form of Eq. (5). For these equations, *x* is the peak resultant rotational acceleration, *v* is the location parameter, σ is the scale parameter, *I*_o_ is the modified Bessel function of the first kind, and *Q*_1_ is the Marcum Q-function. Rician distribution parameters were estimated using a maximum likelihood technique.

4 $$ r_{\text{pdf}} = \frac{x}{{\sigma^{2} }}e^{{\left( {\frac{{ - \left( {x^{2} + v^{2} } \right)}}{{2\sigma^{2} }}} \right)}} I_{\text{o}} \left( {\frac{xv}{{\sigma^{2} }}} \right) $$

5 $$ r_{\text{cdf}} = 1 - Q_{1} \left( {\frac{v}{\sigma },\frac{x}{\sigma }} \right) $$

Then, the relationship between resultant rotational acceleration and resultant rotational velocity was determined. For this sub-analysis, only impacts with peak linear accelerations greater than 40 g in the 6DOF dataset were considered. Impacts were limited to 40 g for data reduction purposes, as each impact’s acceleration traces were visually inspected so that the rotational acceleration pulse of interest could be examined and peak values identified. Furthermore, 40 g is well below typical linear accelerations associated with concussion.^45^ To determine change in resultant rotational velocity, rotational acceleration about each individual axis of the head was numerically integrated with respect to time throughout the entire acceleration trace. Resultant rotational velocity was then calculated. Once peak rotational acceleration and peak change in rotational velocity were identified for each impact, a linear regression analysis between the two parameters was performed using a least squares technique. The regression model was constrained so that a rotational acceleration of 0 rad/s^2^ resulted in a rotational velocity of 0 rad/s. Equation (6) displays the regression model, where ω is resultant rotational velocity, α is resultant rotational acceleration, and *m* is the inverse slope parameter. Equation (6) was used to estimate resultant rotational velocities associated with the peak rotational accelerations in the HIT System dataset.

6 $$ \omega = \frac{\alpha }{m} $$

An injury risk function for resultant rotational acceleration was developed. To do this, published injury incidence rates for game participation were used to weight the sub-concussive and concussive head acceleration distributions. For collegiate athletes, there are 5.56 concussions per 1000 athletic exposures, where an athletic exposure is defined as one athlete participating in at least one play of one game or practice.^1^ To relate the number of concussions to the number of sub-concussive impacts, it was assumed that the median player experiences 16.3 impacts per game.^7^ For collegiate athletes, 5.56 concussions per 1000 games played with 16.3 impacts per game per player can be expressed as an injury incidence rate of 0.341 concussions per 1000 impacts. It is important to note that current research suggests that as many as 53% of concussions go unreported.^30^ This underreporting rate was applied to the calculated injury incidence rate, resulting in 0.726 concussions per 1000 impacts for collegiate athletes.

Next, estimated injury incidence rates were used to combine the sub-concussive and concussive head acceleration distributions in order to have a sub-concussive to concussive impact ratio that reflects previous studies. A logistic regression analysis based on the weighted sub-concussive and concussive head acceleration distributions was used to express risk as a function of rotational head acceleration. Equation (7) displays the risk function, where α and β are regression coefficients. The regression coefficients were determined using a generalized linear model technique.

7 $$ {\text{risk}} = \frac{1}{{1 + e^{{ - \left( {\alpha + \beta x} \right)}} }} $$

## Results

A total of 300,977 head impacts were recorded and analyzed in this study. Of these impacts, 286,636 head impacts were recorded using the HIT System and 14,341 head impacts were recorded using the 6DOF measurement device. A total of 57 concussions were compiled for this analysis. Of the impacts to the front or back of the helmet that resulted in primarily sagittal plane rotation, there were 193,465 sub-concussive impacts (67.5% of total sub-concussive impacts) and 33 concussive impacts (57.9% of total concussive impacts). Of the impacts to the sides of the helmet that primarily resulted in coronal plane rotation, there were 49,645 sub-concussive (17.3%) and 7 concussive (12.3%) impacts. There were 43,526 sub-concussive (15.2%) and 17 concussive impacts (29.8%) to the top of the helmet recorded with the HIT System, which were analyzed separately because they are primarily linear events.

**Figure 2:**
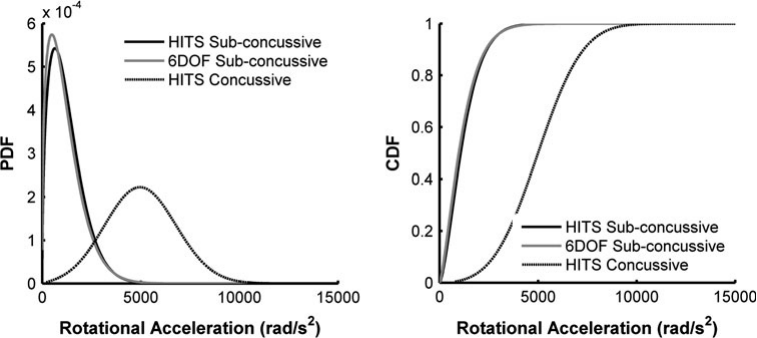
Weibull distributions were fitted to resultant rotational head acceleration for sub-concussive impacts recorded with the HIT System and 6DOF measurement device. A Rician distribution was fitted to resultant rotational head accelerations for concussive impacts recorded with the HIT System. Probability density functions (left) and cumulative density functions (right) are displayed for each distribution fit

**Figure 3:**
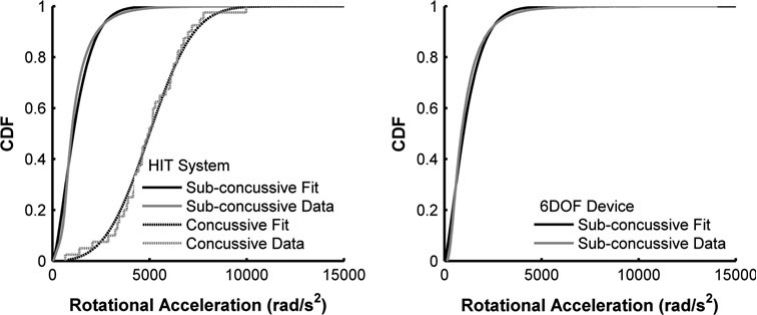
Comparison of the empirical cumulative density functions to the fitted cumulative density functions suggest good fits for both the HIT System datasets (left) and 6DOF measurement device dataset (right)

The sub-concussive impact distribution recorded with the 6DOF measurement device was right-skewed with a 25th percentile rotational acceleration of 531 rad/s^2^, median rotational acceleration of 872 rad/s^2^, and 75th percentile rotational acceleration of 1447 rad/s^2^ (average rotational acceleration of 1158 ± 972 rad/s^2^). The sub-concussive impact distribution recorded with the HIT System was right-skewed with a 25th percentile rotational acceleration of 682 rad/s^2^, median rotational acceleration of 981 rad/s^2^, and 75th percentile rotational acceleration of 1506 rad/s^2^ (average rotational acceleration of 1230 ± 915 rad/s^2^). Concussive impacts were normally distributed with a 25th percentile rotational acceleration of 4026 rad/s^2^, median rotational acceleration of 4948 rad/s^2^, and 75th percentile rotational acceleration of 6209 rad/s^2^ (average rotational acceleration of 5022 ± 1791 rad/s^2^). No concussive impacts were recorded with the 6DOF measurement device during the measurement interval. Figure 2 displays the probability density functions and cumulative density functions for all sub-concussive and concussive impacts with relation to rotational acceleration. Figure 3 displays that the empirical cumulative density functions closely match the fitted cumulative distributions for each dataset. Table 1 displays the parameter estimates for each distribution fit (Eqs. 2–5).

**Table 1:** Distribution fitting parameter estimates for Weibull (Eqs. 2 and 3) and Rician (Eqs. 4 and 5) distributions

	Weibull		Rician	
	α	β	σ	ν
Sub-concussive HITS	1369.8 (1.976)	1.4875 (0.002)	–	–
Sub-concussive 6DOF	1277.6 (8.283)	1.3670 (0.008)	–	–
Concussive HITS	–	–	1863.2 (329.5)	4626.2 (235.1)

The standard error for each parameter estimate is in parentheses

**Figure 4:**
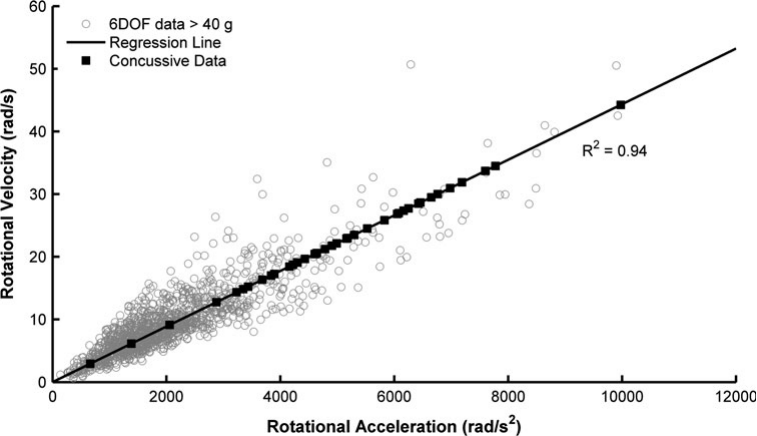
Linear regression relating peak rotational acceleration to peak rotational velocity for 1285 impacts recorded using the 6DOF measurement device that had peak linear accelerations greater than 40 g. Using this model, rotational velocities were estimated for concussive impacts recorded using the HIT System

A total of 1285 impacts were recorded with the 6DOF measurement device that had peak linear accelerations greater than 40 g and were used to quantify the relationship between rotational acceleration and rotational velocity. Peak rotational acceleration and peak rotational velocity correlated strongly (*R* ^2^ = 0.94) in the 6DOF dataset, proving to be a linear relationship (Fig. 4). The inverse slope parameter (*m*) in Eq. (6) was determined to be 225.5 with nominal units of s^−1^. Using Eq. (6), rotational velocities were estimated for concussive impacts from peak rotational acceleration. Table 2 displays the rotational velocities associated with descriptive rotational accelerations of note.

**Table 2:** Descriptive statistics of rotational accelerations distributions with associated rotational velocities

	Descriptive statistics									
	25th Percentile		Median		75th Percentile		95th Percentile		Average	
	α	ω	α	ω	α	ω	α	ω	α	ω
Sub-concussive HITS	682	3.0	981	4.4	1506	6.7	2975	13.2	1230	5.5
Sub-concussive 6DOF	531	2.4	872	3.9	1447	6.4	2997	13.4	1158	5.1
Concussive HITS	4026	17.9	4948	21.9	6209	27.5	7688	34.1	5022	22.3

α is rotational acceleration with units rad/s^2^; ω is rotational velocity with units rad/s

**Figure 5:**
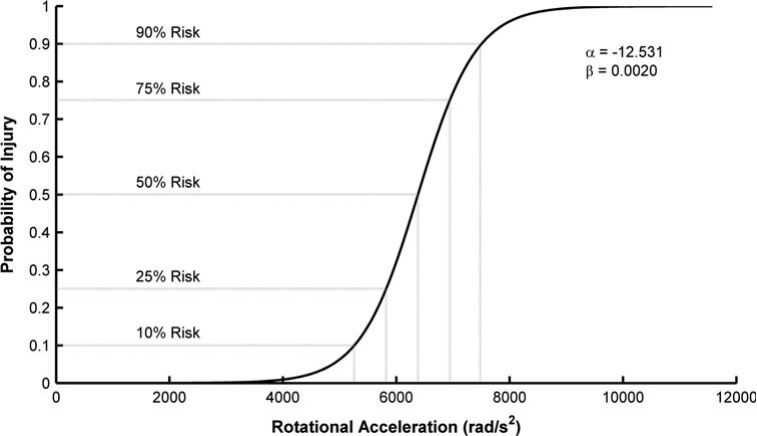
Injury risk as a function of peak resultant rotational acceleration. Parameter estimates for Eq. (7) and nominal injury risk values are superimposed on the plot

Figure 5 displays the probability of concussion as a function of peak rotational acceleration. The risk function (Eq. 7) parameter estimates were determined to be −12.531 for α and 0.002 for β. Table 3 displays rotational accelerations and rotational velocities for nominal injury risk values.

**Table 3:** Rotational accelerations and rotational velocities associated with nominal injury risk values

Nominal injury risk	Rotational acceleration (rad/s^2^)	Rotational velocity (rad/s)
10%	5260	23.3
25%	5821	25.8
50%	6383	28.3
75%	6945	30.8
90%	7483	33.2

Rotational accelerations of lower magnitudes were observed with impacts to the top of the helmet. Sub-concussive impacts to the top of the helmet recorded with the 6DOF measurement device were right-skewed with a 25th percentile rotational acceleration of 346 rad/s^2^, median rotational acceleration of 595 rad/s^2^, and 75th percentile rotational acceleration of 1057 rad/s^2^ (average rotational acceleration of 845 ± 798 rad/s^2^). Sub-concussive impacts to the top of the helmet recorded with the HIT System were right-skewed with a 25th percentile rotational acceleration of 266 rad/s^2^, median rotational acceleration of 446 rad/s^2^, and 75th percentile rotational acceleration of 768 rad/s^2^ (average rotational acceleration of 615 ± 565 rad/s^2^). Concussive impacts to the top of the helmet recorded with the HIT System had a 25th percentile rotational acceleration of 617 rad/s^2^, median rotational acceleration of 1822 rad/s^2^, and 75th percentile rotational acceleration of 3673 rad/s^2^ (average rotational acceleration of 2192 ± 1790 rad/s^2^).

## Discussion

These data provide, for the first time, an estimate of rotational acceleration tolerance derived from direct acceleration measurements from instrumented human volunteers. The rotational acceleration distributions for the 6DOF measurement device and the HIT System were in good agreement. The small differences between the distributions can be attributed to the effect of varying head impact exposures for different football positions among instrumented players. The 6DOF dataset was collected from lineman, because these subjects wear larger helmets that could accommodate the 6DOF measurement device. The HIT System dataset was collected from lineman and skill players. Recent research has shown that lineman sustain impacts more frequently at lower magnitudes relative to skill players.^7^ The minimal difference in distributions between the two datasets suggests that the HIT System was capable of accurately quantifying the head impact exposure of rotational acceleration experienced by the instrumented football players.

While rotational acceleration could be reasonably calculated with the HIT System, a rotational acceleration without a rotational velocity is difficult to interpret with relation to injury tolerance. A rotational velocity associated with a rotational acceleration provides information about the temporal component of the acceleration pulse. Rotational head accelerations of great magnitudes can be tolerable over very short durations; however, as duration increases, tolerance decreases.^39^ Moreover, rotational velocity was of particular interest in this study because it has been shown to have a stronger correlation with relative brain motion than any other kinematic parameter.^19^,^21^ Computational studies have also found rotational velocity to be a predictor of the strain response when modeling real-world head impacts that were experimentally recorded from football players.^51^ Peak rotational acceleration and peak rotational velocity in the 6DOF dataset were strongly correlated. The strong correlation between the two parameters suggests that head acceleration pulses as a result of head impacts in football are similar in duration and acceleration shape. The linear regression model was used to determine the average rotational velocity associated with peak rotational acceleration at sub-concussive and concussive severities.

**Figure 6:**
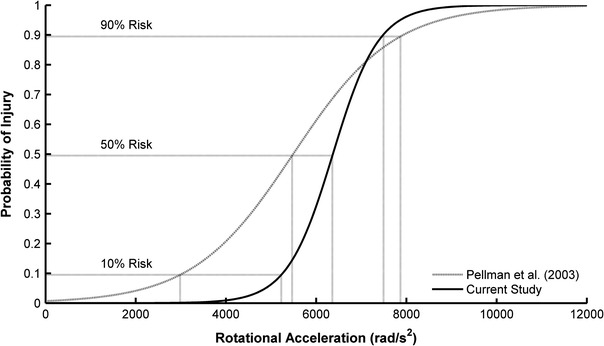
Comparison of the concussion risk curve generated in this study to that of Pellman *et al.* 42 Nominal injury values of 10, 50, and 90% are emphasized to display differences between the two curves at varying severities

Injury risk was assessed as a function of rotational acceleration through an analysis of a large dataset of head impacts. Acceleration distributions for sub-concussive and concussive impacts were weighted to reflect a defined ratio between sub-concussive and concussive impacts. The distribution weighting techniques utilized published concussion incidence rates and considered the under-reporting of concussions, which is a problem of increasing concern.^2^,^10^,^30^,^55^ It should be noted that the risk curve generated in this study may be conservative (i.e., over-estimate risk). This is for two main reasons: (1) the highest reported injury incidence rate from the literature was used for the relative weighting of sub-concussive and concussive impacts, and (2) the risk curve accounts for the under-reporting of concussive injuries. Pellman *et al.* 42 generated injury risk curves for concussion from reconstructed NFL impacts using Hybrid III ATDs. In that study, the average concussive impact (*n* = 25) had a rotational acceleration of 6432 rad/s^2^ and rotational velocity of 36.5 rad/s. The average sub-concussive impact (*n* = 33) had a rotational acceleration of 4028 rad/s^2^ and rotational velocity of 26.1 rad/s. Figure 6 compares the injury risk curve derived from the NFL data for rotational acceleration to the risk curve produced in this study. In comparison to the risk curve generated in this study, the NFL risk curve over-predicts injury risk at lower acceleration magnitudes (risk < 50%) and produces similar values at higher acceleration magnitudes (risk > 50%). The differences between the two risk curves can partially be attributed to the NFL data being biased toward concussive impacts. Furthermore, the NFL data were based on reconstructions from game film using Hybrid III ATDs. While the Hybrid III is often used to evaluate sports injury scenarios in the laboratory,^47^,^49^ the neck of the Hybrid III has limited biofidelity. The Hybrid III ATD reconstructions produced similar peak accelerations for concussive impacts, but generated higher rotational velocities. The temporal response of the Hybrid III neck to head impact is elongated due to its low stiffness.^18^ Although the use of the Hybrid III has caveats, it remains a valuable tool when collecting data from humans is not feasible.

**Figure 7:**
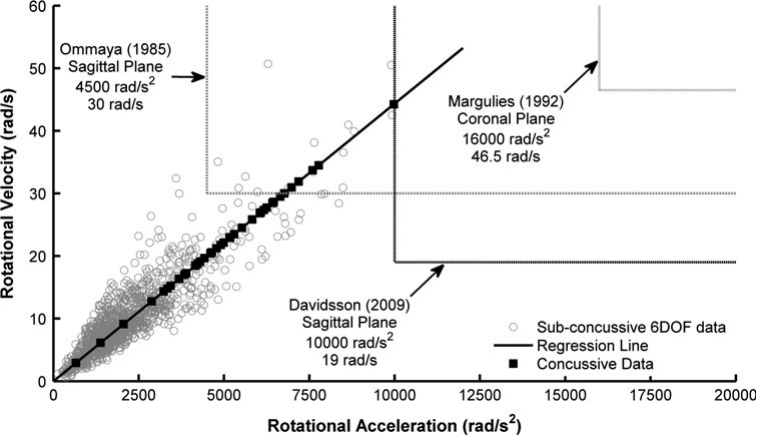
Comparison of sub-concussive and concussive data collected from football players to DAI thresholds derived from animal data that were scaled to reflect human data

Previous studies have generated rotational kinematic thresholds from scaled animal data for DAI. Although DAI is a more severe injury than the sports-related concussion injury analyzed in this study, there is value in comparing results. Ommaya^39^ utilized a primate model and suggested an injury threshold of 4500 rad/s^2^ when rotational velocity is less than 30 rad/s for sagittal plane rotation of the head. Additionally, Davidsson *et al.* utilized a rat model and suggested a threshold of 10,000 rad/s^2^ with a rotational velocity of 19 rad/s for rearward sagittal plane rotation.^8^ For coronal plane rotation, Margulies and Thibault^27^ utilized a primate model and suggested a threshold of 16,000 rad/s^2^ with a rotational velocity of 46.5 rad/s. Figure 7 compares these published thresholds for DAI to the data collected from football players. The kinematics of these experiments had a negligible linear component, as they were designed to invoke pure rotation of the head. While theoretically possible, this phenomenon is likely rarely experienced in the real-world because the high magnitude accelerations require head contact to occur.^23^ No head impact measured in football players was comprised of pure rotation. Moreover, these animal studies limited rotation to a single plane of the head, while the impacts measured from football players involved rotation in all three planes of the head simultaneously. With that said, the average concussive values of 5022 rad/s^2^ and 22 rad/s generated in this study are most similar to that of Ommaya.^39^ However, the criteria derived from primate data were proposed to predict prolonged unconsciousness greater than 6 h and neuropathologic findings of DAI. Ommaya’s criterion was self-admittedly speculative for injury to humans due the scaling techniques used to transform the rhesus monkey data to human data.^39^,^41^ Similar caution should be exercised when drawing conclusions based on injury thresholds derived from Margulies and Thibault^27^ and Davidsson *et al.* 8

**Figure 8:**
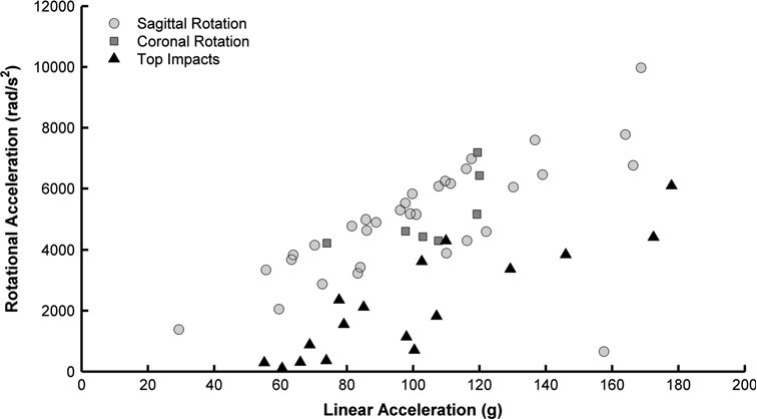
Linear and rotational accelerations for concussive impacts grouped by impact mode. Impacts to the top of the helmet had similar peak linear accelerations and lower rotational accelerations than other impact locations

While the 6DOF measurement device was used to measure rotational acceleration, Eq. (1) was used to calculate rotational acceleration for impacts recorded with the HIT System. Equation 1 calculates rotational acceleration from the resultant linear acceleration along the anterior–posterior and medial–lateral axes of the head and a combined variable representing the average inertial properties of the head and average direction of force. Since rotational acceleration for the HIT System is determined from the acceleration vector of the head CG in the transverse plane, this analysis is insensitive to transverse rotation and only considers sagittal and coronal plane rotation. Of the impacts recorded, 67.5% were to the front or back of the helmet; indicating that the majority of impacts were dominated by sagittal plane rotation. These data are consistent with those previously reported.^7^,^33^ Notably, linear acceleration along the inferior–superior axis of the head is not considered in Eq. (1), although top impacts were included in its derivation. Impacts that had the largest accelerations along this axis likely had little rotation due to the impact force being transmitted through (or near) the head CG and neck. For this reason, impacts to the top of the helmet were separated from the distribution and risk analyses, as this study focuses on the rotational kinematics. Figure 8 compares the linear and rotational accelerations associated with concussion for impacts that were generalized into three groups: sagittal rotation, coronal rotation, and impacts to the top of the helmet. While, throughout the course of a season, a player experiences fewer impacts to the top of the helmet than to the front and back of the helmet, the number of concussions per impact to the top location is the greatest. This is a result of impacts to the top of the helmet being greater energy impacts, likely due to a player purposely leading an impact with his helmet.^45^ Table 4 compares the average linear acceleration, rotational acceleration, and rotational velocity for each of the three groups. Although the linear accelerations for each impact mode were very similar, rotational kinematics for impacts to the top of the helmet were substantially lower than impacts to the front, back, or sides of the helmet. This supports the notion that both linear and rotational components of acceleration contribute to concussion.^40^

**Table 4:** Average concussive linear acceleration and rotational kinematics for impacts that were either primarily sagittal plane rotation (front and rear impact locations), primarily coronal plane rotation (side impact locations), or to the top of the helmet

	Number of concussions	Linear acceleration (g)	Rotational acceleration (rad/s^2^)	Rotational velocity (rad/s)
Sagittal plane rotation	33	102.7 ± 33.6	4986 ± 1909	22.1 ± 8.5
Coronal plane rotation	7	105.8 ± 16.6	5192 ± 1166	23.0 ± 5.2
Impacts to helmet top	17	100.6 ± 37.1	2192 ± 1790	9.7 ± 7.9

Linear and rotational acceleration have traditionally been examined independently of one another, even though both can contribute to brain injury.^23^,^40^ This is largely due to linear acceleration and rotational acceleration being correlated to different injury mechanisms. Studies have suggested that linear acceleration is correlated to the intracranial pressure response,^21^,^52^,^54^,^56^ and that rotational kinematics are correlated to the strain response of the brain.^24^,^51^,^52^ Brain injuries due to linear acceleration are typically focal in nature, while brain injuries due to rotational acceleration are typically diffuse in nature, but can also produce focal damage.^39^ Ommaya^39^ suggested the use of dual criteria when predicting brain injury due to head kinematics: his own rotational kinematic criterion and the maximum strain criteria,^32^,^50^ which is based on linear acceleration. In this same light, several researchers have suggested that combined linear and rotational kinematic parameters are likely to have the greatest predictive capabilities of concussion.^15^,^36^ With the increased understanding of injury risk related to single biomechanical parameters, more work should be conducted investigating the combined role of linear and rotational kinematics in producing injury.

The kinematics associated with concussion appear to be clearly defined as a non-zero normal distribution, which indicates there is a correlation between mechanical input and clinical outcome. However, there were many impacts with accelerations at concussive levels that did not result in injury. This suggests that individual differences might play an important role in determining human tolerance to concussion. Although these factors need further clarification, some potential contributors include whether the impact was anticipated or not, as well as functional polymorphisms in genes modulating response to neurotrauma.^29^ There may also be additional biomechanical predictors of brain injury than head kinematics. By using head kinematics as input to finite element head models, the tissue level response of the brain can be quantified, and the strain or pressure response (or any other parameter of interest) can be used to assess injury.^24^,^51^,^56^ However, before this is possible, the best injury predictors must be determined and validated using injury data, such as the field data presented in this study. Unfortunately, these predictors are likely to be model-specific, as each model may find a different parameter that best predicts injury.

This study has several limitations. First, it should be noted that linear acceleration was measured using the HIT System and rotational acceleration was calculated from a linear acceleration vector, the inertial properties of the head, and an average direction of force. Although rotational acceleration was not directly measured, the calculation provides a good estimate. Second, there is measurement error associated with both the HIT System and 6DOF measurement device. However, the average errors of these devices are on the order of 1–4%. While there may be greater errors associated with individual data points, these errors are of little consequence when working with the overall data distributions. Third, many concussions sustained while participating in football are unreported or undiagnosed. This study makes an attempt to account for unreported concussions in our injury incidence calculation, but the under-reporting of concussions may bias our data. Furthermore, potential variation in injury and injury diagnosis is not accounted for. These factors may explain some of the variation observed in the biomechanical data. Fourth, this study examines data across an entire cohort and did not account for variations in head impact exposure associated with individual players. It is unclear how that analysis would influence tolerance levels. Moreover, no attempt was made to quantify the effects of cumulative head impacts, which may or may not affect individual tolerance, and concussion injuries were associated with a single impact. Finally, although every impact was composed of linear and rotational kinematics, this study investigates rotational kinematics independent of linear acceleration. More work is needed investigating the combined contribution of linear and rotational kinematics to brain injury.

The significance of this study lies within methods that collect biomechanical head impact data from humans at potentially injurious severities and pairing these data with clinical diagnosis. Large sub-concussive and concussive datasets were analyzed and characterized. This study addresses the limitations of earlier experiments, in that it is the first to present data on 57 concussions that were measured directly from human subjects. Valuable insight to the rotational kinematics associated with concussion in humans has been presented. With an increased understanding of the kinematics associated with injury, engineering analyses can be used to evaluate and influence product design to reduce injury incidence.^46^,^47^
